# Back School Postural Education Program: Comparison of Two Types of Interventions in Improving Ergonomic Knowledge about Postures and Reducing Low Back Pain in Adolescents

**DOI:** 10.3390/ijerph18094434

**Published:** 2021-04-22

**Authors:** Beatriz Minghelli, Carla Nunes, Raul Oliveira

**Affiliations:** 1 School of Health Jean Piaget Algarve, Piaget Institute, 8300-025 Silves, Portugal; 2 Research in Education and Community Intervention (RECI), 3515-776 Viseu, Portugal; 3 National School of Public Health, NOVA University of Lisbon, 1600-560 Lisbon, Portugal; CNunes@ensp.unl.pt; 4 Interdisciplinary Centre for the Study of Human Performance (CIPER), Human Kinetics Faculty, University of Lisbon (UL), 1499-002 Lisbon, Portugal; roliveira@fmh.ulisboa.pt

**Keywords:** back school program, low back pain, literacy, adolescent, backpack

## Abstract

This study aimed to compare the impact of two Back School Postural Education Programs on improving ergonomic knowledge of postures adopted at school and home, as well as on reducing low back pain (LPB) in adolescents. The sample was constituted by 153 students, aged 10–16 years, with 96 (62.7%) girls, divided into 2 intervention groups (GA, GB). Two tests (theoretical and practical) and LBP questionnaire were applied 1 week before and 1 year after the end of the program. In GA, three sessions were performed for each class separately, on theoretical and practical issues, lasting 45 min and at intervals of 1 week, and in GB, only one theoretical session (90 min) was given to all students. Statistically differences on GA were obtained between the values 1 week before and after 1 year of evaluation in both theoretical and practical tests (*p* ≤ 0.001). In GB, only the values of the practical test present a statistical difference (*p* ≤ 0.001). GA obtained higher values on both tests after 1 year of follow-up compared with GB (*p* ≤ 0.001). The number of students with LBP decreased on GA (*p* ≤ 0.001). The program with longer duration, higher weekly frequency, and more practical and individualized character promotes better effects.

## 1. Introduction

Our posture requires a continuous, precise, and complex interaction of action and perception systems and higher-level processes [1]. Adolescents can be predisposed to the development of musculoskeletal disorders due to the inadequate and/or prolonged static postures adopted in the classroom, the inadequate school furniture, the inadequate transport of the school backpack, and the excess of weight. These risk behaviors related to posture are associated with periods of a growth spurt, which contribute to the development of postural changes and low back pain (LBP) in adolescence [2,3,4,5,6,7].

LBP is a multifactorial condition that has become a growing and serious public health problem in adolescents, and approximately 80% to 85% of the LBP episodes have no known cause [8,9]. Some studies verified an association between LBP and adoption of some postures [9,10]. Sjolie’s [11] study revealed that the most common situations that caused LBP were manual work (70%) and sitting at school (48%), and Grimmer and Williams [4] also found associations between LBP and the amount of time spent in a seated posture. Minghelli et al. verified that students who sit with their spine in the wrong position had 3.24 more chances to have LBP [12].

Considering that postural changes and LBP can be associated with the environment and lifestyles (modifiable risk factors), it is necessary to carry out intervention actions aimed at health promotion and prevention. The back school program emerges to promote knowledge and health conditions in these specific areas, optimizing the technical and personal skills of teachers and students and developing individual and collective health potential through increased literacy and empowerment.

Several studies [13,14,15,16,17,18,19] have verified the effectiveness of a back school program in improving students’ posture and knowledge about ergonomic issues; however, there is diversity in the intervention programs applied and still no specific guidelines established. In addition, schools have a curriculum program to fulfil, and cannot dispense many hours for the development of such programs. Dolphens et al. [16] performed a 6-week school-based back education program, for students aged 9- to 11-years old, at 1-week intervals, and the results showed an increase in back care knowledge, but the intervention did not change spinal care behaviour. Cardon et al. [15] evaluated students in fourth and fifth grade in elementary school and verified that the back care education program constituted by six sessions promoted improvements in knowledge test and practical assessment. Regarding national studies, Minghelli [20] verified that a school physiotherapy programs, constituted by only one session, promoted an increase in health literacy in students aged 12–19 years old. It can be seen that the number of sessions varied between these studies.

In this context, this study had the following objectives: To evaluate and to compare the impact of two Back School Postural Education Programs (with different number of sessions) on improving ergonomic knowledge of postures adopted at school and at home, as well as reducing non-specific LPB in adolescents (1-year follow-up).

## 2. Materials and Methods

The study design was longitudinal. The study was approved by the Piaget Institute’s Research Unit RECI (Research in Education and Community Intervention), by National School of Public Health (ENSP, NOVA University of Lisbon) and by Dr. Garcia Domingues School Direction. Written informed consent was obtained from all parents or guardians of the students.

### 2.1. Population

The population included students from the public school E.B. 2,3 Dr. Garcia Domingues, in Silves, southern Portugal. For logistics reasons, we chose the 5th and 7th grades.

In the school year analyzed, there were 5 classes of the 5th grade with 97 students enrolled and 5 classes of the 7th grade with 101 students, totaling 10 classes with 198 students.

The population was divided into 2 intervention groups (GA, GB). Six classes were chosen for the intervention group A (GA), with this being 3 classes of the 5th grade and 3 of the 7th grade, totaling 118 students; for intervention group B (GB), 4 classes were selected, with this being 2 classes from the 5th grade and 2 from the 7th grade, with a total of 80 students. The division of the population into groups took into account the impossibility of dividing the classes. In other words, students in the same class could not be separated, as they should watch the intervention together. Given that the acquisition of knowledge is different in the 5th and 7th years of schooling, we could not have a group with more classes in the 7th year, for example.

Inclusion criteria cumulatively involved students of both sexes, of any age, whose parents (or legal responsible) authorized study participation, and who were present on the days of the evaluations. Students of GA who did not attend at least 2 sessions of Back School and Postural Education Program and those of GB who did not attend the session were excluded from the study. The intervention groups will be explained later.

### 2.2. Measurement Instrument

The measurement instruments included a theoretical and a practical test, a questionnaire, and a scale. All instruments used were applied 1 week before and 1 year after the end of the program for the both groups (GA and GB). In all assessments, the principal investigator was always present for both execution and supervision, ensuring that the rules and instructions given were homogenous. The group of evaluators has always been the same in all evaluations, having been previously trained.

#### 2.2.1. Theoretical Test

A theoretical test was applied to students to assess the level of theoretical knowledge about correct posture, dynamic and static, and the distribution of school material, method of transport, and amount of weight to be carried in the backpack; this test was constituted by 13 multiple-choice questions and the answer options were in the form of figures to facilitate the perception of the requested activity.

This theoretical test was adapted from several studies that included questions about the use of mobile devices and sleep posture; however, the questions about spine anatomy were removed [15,18].

Questions on the final questionnaire involved the best way to: (1) Lift a heavy box off the floor; (2) carry your shopping bags; (3) carry your backpack; (4) carry a heavy box; (5) move a heavy box; and (6) put your school supplies in your backpack; the best position: (7) In which to sleep; (8) for your back when sitting; (9) for your feet when sitting; (10) for your back for watching TV and/or playing games; (11) the best standing posture; (12) the best posture to use your mobile phone; and (13) the maximum weight you should carry in your school bag.

Each correct question had a quote of 1 value and for each wrong question, and it was assigned a score of −1. In this way, the maximum score of the test was 13 and the minimum was −13.

#### 2.2.2. Practical Test

The evaluator requested the tasks that should be performed, without any kind of demonstration. Each student performed the test individually.

The practical test used was adapted from other studies [14,15,17,18] and was composed by 15 items divided in 5 tasks. For each task, a standard posture was defined that was considered as the correct posture, and for each correctly executed item, a score of 1 value was given, totaling a maximum of 15 values.

**Figure 1:**
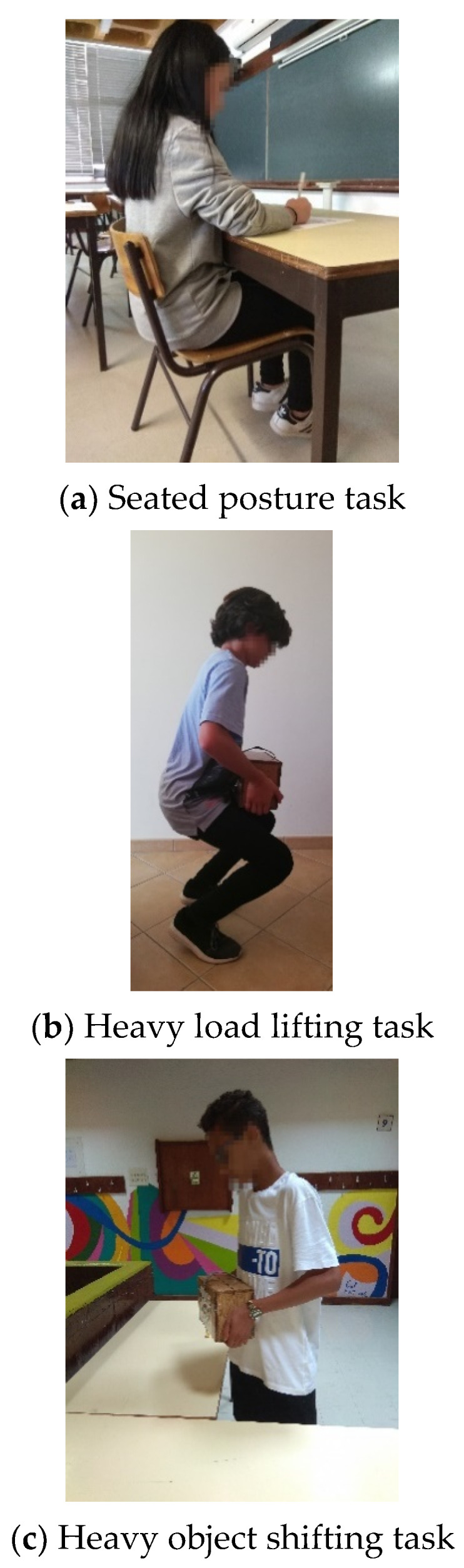
Representation of (**a**) seated posture, (**b**) heavy load lifting, and (**c**) heavy object shifting tasks.

In the first task, called “Seated Posture”, the student was asked to sit in a chair and write some data. This first task had as objective to evaluate the seated posture during a task of writing. The standard posture consisted of the student being seated with the spine supported on the back, with the gluteal region close to the back, with an approximate angle of 90° between the thigh and the leg and with the feet resting on the floor, if the size of the chair so allow (4 items evaluated) (Figure 1a).

For the second task called “Heavy load lifting”, the student was asked to lift from the ground a 6.5 kg wooden box, which mimicked the dimensions and weight of a regular 6 L package of milk and returned to put it on the other table. The objective of using a 6 L of milk pack was to simulate an object easily found for sale in supermarkets (familiar to the student) and which has only one transport handle in the center of the package. The objective was to verify if the students transported this package without using the single handle of the package, because if he/she did, the load would be distributed only to one side of the body. Three items were evaluated in this task: Spine position should be in extension (should not perform flexion and/or rotation); students should bend their knees to catch the object; and the object should be close to the body when going to stand, without using the single handle of the package (Figure 1b).

For the next task called “Light Loading”, the student picked up a pen of 10 g (light object) from the floor and placed it on a table. Two items were evaluated in this task: Spine position should be in extension (should not perform flexion and/or rotation); and students should bend their knees to catch the pen.

On the fourth task called “Heavy Object Shift”, the student was asked to move the wooden box to another table next to it. In this task, 2 items were evaluated: The position of the spine, avoiding rotation movement; and the object should be close to the body. To avoid spine rotation, the student should move in a block, moving his/her feet (Figure 1c).

Finally, in the task called “Transportation, storage and weighing of the school backpack” 4 items were evaluated: The student was asked which is the most frequent way of transport of his/her backpack (should use the 2 handles one on each shoulder); the adjustment of the backpack to the body of the student was verified (the backpack should be adjusted very close to the back); how the material was distributed was verified (lighter materials, such as snacks and cases, should be further away from the back and the heavier material, such as books and notebooks, should be closer to the back); and whether the weight of the backpack was adequate to the weight of the student was verified. The backpack and student were weighed using a SECA 780 digital scale with a capacity of 150 kg and a precision of 100 g. A backpack was classified as having excessive weight if it was more than 10% of the individual’s body weight [3].

#### 2.2.3. Low Back Pain Questionnaire

The low back pain questionnaire used was the same as in Minghelli et al.’s [10] study, but the questions related to the evaluation of students’ posture habits were excluded.

The questionnaire included questions about the biological characteristics of the students (gender and the student’s age), and about the occurrence of LBP at the moment of application of the questionnaire and in the last 12 months.

LBP was characterized by the presence of symptoms in the lumbar region, between the last ribs and the lower gluteal folds, that lasted for at least 24 h and include pain, muscular tension, or stiffness [21].

The questionnaire was applied in the form of an interview by the evaluator.

Note: The theoretical and practical tests and the Low back pain questionnaire are available at the end of this article. These instruments are presented in Portuguese as it was not yet validated to other languages.

### 2.3. Back School Postural Education Program

These interventions aimed to improve the mechanics and the static and dynamic body posture during the daily tasks at school and home.

The sessions of both group interventions (GA and GB) included the following topics: Spine anatomy; spinal joint physiology (spinal movements and intervertebral disc mechanics); postural changes and spinal pathologies; ergonomic analysis of sitting posture (during the writing and watching a class); sleeping; getting out of bed; standing; lifting and transporting objects; transporting the backpack; the distribution of the material in the backpack; consequences of adopting an incorrect and/or prolonged posture; the importance of intervals after maintaining the static posture; and exercises to be included in these intervals.

The differences between the two intervention programs including the number of sessions, the duration, the number of students per session, and the most practical and individualized intervention.

**Figure 2:**
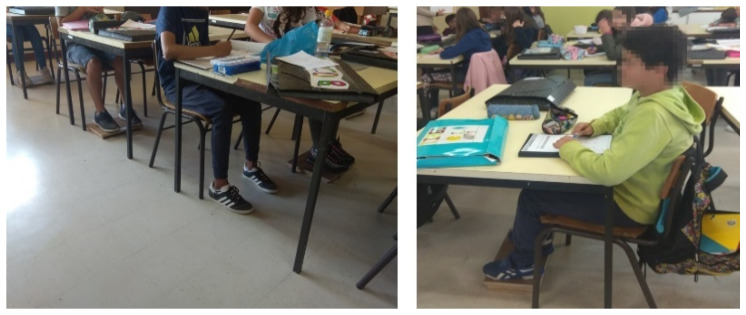
Use of wooden boxes for feet support.

In GA, 3 sessions for each class were performed separately, both of a theoretical and practical nature, lasting 45 min and at intervals of 1 week. In the third session, each student was individually analyzed and corrected for the sitting position and, if necessary, changes were made in the school environment related to furniture, to meet the requirements of correct posture (for the smaller students, wooden boxes were made so that they could support the feet) (Figure 2). Besides that, each student’s backpack was assessed for the adjustment of the backpack close to the student’s back, backpack weight, and distribution of school supplies.

For the classes in GB, only 1 theoretical session was given to all students in this group (*n* = 80), in the school auditorium, with a duration of 90 min. The items related to the last session in GA, namely the individual evaluation of each student regarding the sitting position and their respective backpack, were not performed in the GB, since that session was held in the auditorium and did not correspond to the ambience of a classroom with tables and chairs. The chairs of an auditorium are different from the chairs in the classroom. Thus, it was not possible to evaluate each student, because they were without tables, and because it was a larger group of students there was no time availability to be individually assessing both the posture and the backpacks.

The sessions in both groups were taught by the same physiotherapist with a large amount of experience in this type of intervention.

### 2.4. Data Analysis

First, descriptive statistical data were obtained regarding all the variables in the study. Posteriorly, statistical inference analysis was performed using the Kolmogorov–Smirnov test (to test for normality).

The Wilcoxon-Mann-Whitney test was applied to compare the two types of intervention (2 independent samples). The Wilcoxon test was used to compare the initial and final evaluations in each intervention groups (paired samples).

Regarding the backpack variable, once the data present a population with a normal distribution, the Paired-Sample T-test was used to compare the initial and final weights of a school backpack. Backpack weight was normalized to student weight and is shown as a percentage (backpack weight/student weight × 100).

Chi-square was used to verify the dependence of the variable presence of low back pain in the pre and post-test.

In all inferential analysis, the statistical significance was 0.05.

Statistical analysis was performed using Statistical Package for Social Sciences (SPSS) software, version 24.0 (IBM Corporation, Armonk, NY, USA).

## 3. Results

**Figure 3:**
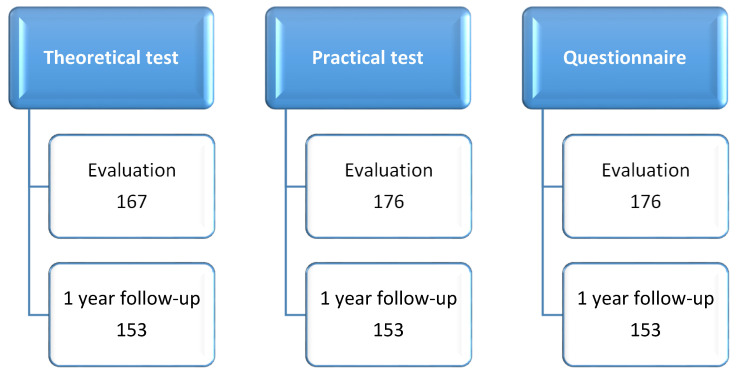
Number of students who performed the evaluations.

Of the 198 students enrolled in the classes, 179 sponsors of education authorized their student’s participation in the program. After 1 year, some students failed, others were transferred to another school, and others were not present on the day of reevaluation. The theoretical test was applied by the teacher in the GB and some students did not perform the test before the beginning of the intervention. The organization chart (Figure 3) shows the number of students who carried out the initial and final evaluations.

The sample of this study was constituted by 153 students, ages between 10 and 16 years old (mean ± standard deviation: 11.59 ± 1.34), being 57 (37.3%) boys and 96 (62.7%) girls. Eighty (52.3%) students were enrolled in 5th grade and 73 (47.7%) in 7th grade (data on the start of the intervention).

Ninety-eight (64.1%) students belonged to the GA and 55 (35.9%) to the GB.

Table 1 presents the mean, standard deviation, minimum and maximum, median, and interquartile range values obtained in both theoretical and practical test 1 week before the program and a follow-up period of 1 year after the intervention. The *p*-value of the Kolmogorov–Smirnov test was ≤0.001, therefore non-parametric approaches were followed. The value of level of statistical significance refers to the comparison of the mean between intervention groups (GA versus GB) in each evaluation period and in each type of test (theoretical and practical).

Table 2 presents the values of level of statistical significance obtained in the comparison of the evaluations before and after the intervention in the two intervention groups. The *p*-value of the Kolmogorov–Smirnov test was ≤0.001, therefore non-parametric approaches were followed.

Table 3 shows the frequency and percentage of variables of the tasks of the practical test before the Back School and Postural Education Program and 1 year after the intervention.

Table 4 shows the values of backpack weight (considering 10% of the student’s body weight), separated by interventions groups and by the period of evaluations. The *p*-value of the Kolmogorov–Smirnov test was ≥0.05, thus a parametric test was used.

Table 5 shows the values of the prevalence of low back pain before and after the intervention program in both groups.

## 4. Discussion

The data of this study allow us to conclude that both types of interventions promoted improvements over an extended period (1 year) in knowledge about ergonomic habits, except for theoretical knowledge in GB.

Our results are similar to those obtained in other studies that verified the effectiveness of back school programs on improving knowledge about back care in young people [14,15,16,17,18,19,22,23] However, when comparing the two types of interventions (135 min (GA) versus 90 min (GB), 3 sessions (GA) versus 1 session (GB), and more practical and individualized (GA) versus theoretical (GB)), there was a more elucidated mean in the values of the evaluations in the students who attended intervention A.

This fact can be explained by the more practical and individualized nature of the GA intervention program (each student was individually corrected). In addition, posture correction was done in the classroom context, with the tables and chairs that students use daily, not with the chairs of an auditorium. Another factor may have also been the number of students attending intervention B: All students (n = 80) were present in the single session, making it more difficult to concentrate on the theme addressed. Furthermore, it was only one session in GB, unlike intervention A that had three weeks in a row (one session per week) where students heard about the postures theme. The problem with doing many sessions, plus assessments, is that you take many classes (five lessons were used in GA) and the school curriculum has to be fulfilled during that school year. One session (GB) would be more convenient for the school and also for the physiotherapist, who can give only one session that encompasses a larger number of classes at the same time.

Regarding the practical test, except the spine posture in the sitting position in both groups and the displacement of a heavy object in GB students, all tasks of the practical evaluation showed improvements after 1 year of the program. This calls attention to the fact that improvements over 90% were made in many tasks performed by GA students and a smaller percentage of performing correct postures in GB, especially in sitting posture when evaluating the spine.

The evaluator observed in practice that many students do not have body consciousness and, therefore, despite knowing in theory what better posture is (after learning in the program), cannot assume a correct posture when adopting the sitting posture.

Posture is still a topic of controversy. It is necessary to clarify that, in the sessions given to the students, a postural pattern was determined as being the most correct, but the importance of adopting several postures seated throughout a school day, alternating the positions, and reducing the maintenance time in the same static position was also clarified. Since time in the sitting position cannot be reduced, students were also encouraged to practice physical activity to compensate for the hours of sedentary posture adopted.

In relation to backpack weight, a statistically significant improvement was observed only in GA. Considering that over 10% of backpacks are overweight [3], even though there was a reduction in backpack weight from 13% to 11% in GA, this overweight problem still exists even after the intervention and some students still carry values far above the recommended (25% in extreme cases). Some studies did not verify statistical significance in the relation between overweight school backpacks and postural changes and/or LBP [24,25,26,27,28]. However, overweight backpacks are a long-standing problem, which, even if proven to not impair the student’s health, causes at least one form of discomfort or muscle fatigue. Having said that, it is still important to develop measures that can reduce their weight, such as the use of digital books, but for this, the school must have the infrastructure to receive this type of technology and all students must also be able to acquire the apparatus for their use.

Considering the LBP issue, there was an improvement in this symptomatology in the GA students in the one-year period, which was the period after the end of the program (36.7% versus 23.5%). At the time of the reevaluation, there was also a lower percentage of students complaining about LBP (16.3% versus 5.1%). The opposite occurred in GB students, in that there was an increase in the number of students who complained of self-reported LBP, except for the period at the time of the evaluations.

Similar results were obtained in Cardon et al.’s [22] study who verified a decrease from 31.9% at the pretest to 23.3% at the 1-year post-test. However, a systematic review of Parreira et al. [29] concluded that there is very-low-quality evidence that back school programs in adults are more effective than no treatment at short, intermediate, or long-term follow-up. However, Zaina and Negrini [30] performed a qualitative check of the RCTs included in this systematic review and verified that more recent studies can provide better results in the long term for pain compared with the absence of treatment, and these authors concluded that programs that involve a more practical component can provide better results of pain at long-term.

LBP may be of multifactorial origin, including mechanical, environmental, and/or psychosocial factors [10,31], making it difficult to conclude that the sessions promoted improvements in their symptomatology; however, the choice of the students for the intervention groups was random, and a more marked improvement in postural knowledge was observed in the students of GA, which may indicate that the adoption of a better posture could lead to an improvement in the symptoms of back pain.

Mwaka et al. [26] verified a significant association between LBP and the time spent sitting, and Minghelli et al. [10] showed that students who sat incorrectly had a 2.49-times greater probability of having LBP (95% CI: 1.91–3.2; *p* < 0.001). Murphy et al. [32] found significant associations between flexed postures and LBP.

The improvement of the performance of the tasks of the practical and theoretical tests does not necessarily mean that the students changed the posture habits; it means that they have acquired a greater knowledge about ergonomic habits and about the health consequences of adopting an incorrect posture.

Future trials should improve methodologic quality and clinical relevance and evaluate the cost-effectiveness of back schools.

## 5. Conclusions

The data of this study allow us to conclude that both types of interventions promoted improvements over an extended period (1 year) in knowledge about ergonomic habits (except for theoretical knowledge in GB). Regarding the LBP symptomatology, only in GA was a decrease of the number of students with the presence of LBP observed after the program; however, this cannot allege that it is an effect of the program, since LBP has a multifactorial origin.

The results between the two types of intervention were different, being better in GA. It has been found that a program with a longer duration, with a higher weekly frequency and a more practical and individualized character, promotes better effects when compared to another program that includes the same programmatic content but is applied once, in fewer hours, and more collectively.

It is necessary to develop strategies that promote the active management of body posture through the creation of a stimulating environment of creativity and a critical sense, not just an intervention aiming at changes in risk factors. Empowerment, capacity, and motivation must be given so that adolescents and the entire school community are responsible for their health choices.

It is believed that if the stimulus continued throughout the intervention year, more benefits could be noted (for example, the teacher corrected the students’ posture during class and those sponsors did the same at home). In this way, the promotion of better body mechanics would continue during class and home activities. Thus, the whole school community needed to be involved in this type of program so that the corrections made at home and in school served as a stimulus to maintain a correct posture. However, this is not always feasible.

Besides, the school policy must include in the information of ergonomic knowledge of postures to the elementary school curriculum prevent musculoskeletal disorders and improve quality of life.

## Figures and Tables

**Table 1:** Values obtained in theoretical and practical tests.

Test	Period	Intervention Groups	Mean ± Standard Deviation	Minimum–Maximum	Median	Interquartile Range	*p*-Value
Theoretical test (−13 to 13)	1 week before	GA	8.7 ± 2.6	2–13	9	4	0.467
		GB	8.9 ± 2.9	5–13	9	4	
	1 year follow-up	GA	11.1 ± 1.9	5–13	11	4	≤0.001
		GB	9.6 ± 2.2	1–13	9	2	
Practical test (0–15)	1 week before	GA	6.4 ± 2.5	1–11	7	3	0.011
		GB	7.6 ± 2.6	1–14	8	3	
	1 year follow-up	GA	12.8 ± 1.8	7–15	13	2	≤0.001
		GB	9.3 ± 3.2	3–15	9	5	

**Table 2:** Comparison between the periods of the evaluations (before and after intervention).

Test	Intervention Group	Period	*p*-Value
Theoretical test	GA	1 week before intervention	≤0.001
		1 year follow-up	
	GB	1 week before intervention	0.62
		1 year follow-up	
Practical test	GA	1 week before intervention	≤0.001
		1 year follow-up	
	GB	1 week before intervention	≤0.001
		1 year follow-up	

**Table 3:** Values of practical test by period of evaluations and by intervention groups.

Tasks of Practical Test		Correct Posture			
		1-Week before Intervention GROUP A	1-Week before Intervention GROUP B	1-Year after Intervention GROUP A	1-Year after Intervention GROUP B
Task 1: Seated posture	Spine position	27 (27.6%)	31 (56.4%)	63 (64.3%)	10 (18.2%)
	Gluteal region position	34 (34.7%)	14 (25.5%)	83 (84.7%)	16 (29.1%)
	Lower limbs position	14 (14.3%)	10 (18.2%)	89 (90.8%)	24 (43.6%)
	Feet position	11 (11.2%)	7 (12.7%)	88 (89.8%)	25 (45.5%)
Task 2: Heavy load lifting	Spine position	26 (26.5%)	26 (47.3%)	83 (84.7%)	33 (60%)
	Knee movement	49 (50%)	33 (60%)	90 (91.8%)	47 (85.5%)
	Object position	22 (22.4%)	9 (16.4%)	80 (81.6%)	24 (43.6%)
Task 3: Light loading	Spine position	43 (43.9%)	31 (56.4%)	95 (96.9%)	42 (76.4%)
	Knee movement	70 (71.4%)	45 (81.8%)	96 (98%)	48 (87.3%)
Task 4: Heavy object shift	Spine position	59 (60.2%)	39 (70.9%)	92 (93.9%)	38 (69.1%)
	Object position	63 (64.3%)	35 (63.6%)	91 (92.9%)	36 (65.5%)
Task 5: Transportation, storage and weighing of the school backpack	Distribution of school supplies	68 (69.4%)	37 (67.3%)	90 (91.8%)	48 (87.3%)
	Backpack transport	75 (76.5%)	43 (78.2%)	88 (89.8%)	48 (87.3%)
	Adjusting the backpack to the student body	51 (52%)	40 (72.7%)	86 (87.8%)	44 (80%)
	Backpack weight	31 (31.6%)	23 (41.8%)	40 (40.8%)	26 (47.3%)

**Table 4:** Values of backpack weight before and after intervention by groups.

Test	Intervention Group	Period	Mean ± Standard Deviation	Minimum–Maximum	Median	Interquartile Range	*p*-Value
Backpack weight (kg)	GA	1 week before	12.8 ± 4.3%	4.0–24.0%	12.6%	20.0%	≤0.001
		1 year follow-up	10.7 ± 4.0%	3.5–24.6%	10.5%	21.1%	
	GB	1 week before	11.3 ± 3.4%	5.0–22.0%	11.0%	18.0%	0.292
		1 year follow-up	10.8 ± 3.3%	3.6–17.4%	9.9%	13.8%	

**Table 5:** Prevalence of low back pain before and after the intervention program.

Intervention Group	Period of Data Collection	Presence of Low Back Pain			
		at the Moment	*p*-Value	12-Month Period	*p*-Value
**GA**	1 week before intervention	16 (16.3%)	*p* = 0.240	36 (36.7%)	*p* ≤ 0.001
	1 year follow-up	5 (5.1%)		23 (23.5%)	
**GB**	1 week before intervention	9 (16.4%)	*p* = 0.067	19 (34.5%)	*p* = 0.387
	1 year follow-up	4 (7.3%)		21 (38.2%)	

## Data Availability

The data obtained in this study were included in an SPSS database. Informed consent and tests applied are on paper filed at my school. None of these documents are available online, only on paper.
